# Use of reverse-transcriptase-based HIV-1 viral load assessment to confirm low viral loads in newly diagnosed patients in Switzerland

**DOI:** 10.1186/1471-2334-14-84

**Published:** 2014-02-13

**Authors:** Beatrice N Vetter, Cyril Shah, Jon B Huder, Jürg Böni, Jörg Schüpbach

**Affiliations:** 1 Swiss National Center for Retroviruses, Institute of Medical Virology, University of Zurich, Zurich, Switzerland

**Keywords:** HIV-1, Viral load underestimation, Nucleic acid test, Reverse transcriptase test, PERT assay

## Abstract

**Background:**

Treatment-naïve patients newly diagnosed with HIV occasionally present with low viral RNA of ≤1’000 copies/ml, raising concerns about viral load underestimation. Because falsely low or undetectable viral loads might lead to inadvertent virus transmission or treatment delays, confirmation of such cases by a sequence-independent viral load test is recommended in Switzerland.

**Methods:**

HIV-1 RNA ≤1’000 cp/ml by Roche’s or Abbott’s tests in patients newly diagnosed from 2010 to 2012 in Switzerland were subjected to viral load testing by the product-enhanced-reverse transcriptase (PERT) assay. These investigations were complemented with repeat and/or alternative viral RNA measurements.

**Results:**

HIV-1 RNA ≤1’000 cp/ml was observed in 71 of 1814 newly diagnosed patients. The PERT assay suggested clinically relevant viral load underestimation in 7 of 32 cases that could be investigated. In four patients, the PERT viral load was 10-1’000-fold higher; this was confirmed by alternative HIV-1 RNA tests. Six of the 7 underestimates had been obtained with meanwhile outdated versions of Roche’s HIV-1 RNA test. In the seventh patient, follow-up revealed similar results for RNA and PERT based viral loads.

**Conclusion:**

PERT assay revealed occasional severe viral load underestimation by versions of HIV-1 RNA tests meanwhile outdated. Underestimation by contemporary tests appears rare, however.

## Background

The quantification of HIV-1 RNA in plasma has become the standard for assessing a patient’s plasma viral load (VL) at the time of diagnosis and throughout the course of the disease. Real-time PCR-based nucleic acid tests (NAT) have a high sensitivity and broad dynamic range [1] and the use of fine-tuned primer/probe sets has made it possible to detect isolates of HIV-1 groups M, N, and O as well as all known subtypes and circulating recombinant forms. However, due to the highly variable nature of HIV, primer/probe binding to the target region occasionally can be sub-optimal, resulting in underestimation of VL [2,3]. It is important to establish such underestimation at the time of diagnosis, as VL monitoring will be used for monitoring disease progression and the success of antiretroviral therapy (ART). Falsely low or even undetectable viral load due to underestimation may impact the interpretation of the success of ART and increase the risk of inadvertent virus transmission. In Switzerland, regulations on HIV confirmation testing issued by the Swiss Federal Office of Public Health (SFOPH) in 2006 therefore request that a VL ≤1’000 cp/ml be confirmed by the product-enhanced reverse transcriptase (PERT) assay, a sequence-independent test that quantifies retrovirus particles based on their content of enzymatically active reverse transcriptase (RT) [4]. This test has been available in our country since 1994, and its sensitivity is comparable to NAT [5,6]. VL based on the PERT assay, or on other RT-based tests, have been shown to correlate well with RNA-based VL over a wide dynamic range (10^2^-10^6^ cp/ml) [6-8].

Here, we analyzed the frequency of NAT-based VL ≤1’000 cp/ml among newly diagnosed, untreated HIV-1 patients in Switzerland and investigated whether such low VL were true or due to underestimation. To this end, we compared viral loads derived from NAT and PERT at the time of diagnosis and complemented these data with repeat and/or alternative NAT measurements. Alternative NAT involved testing in a different primer/probe target region, addressing the possibility of sequence polymorphism-associated failure of viral RNA detection.

## Methods

### Study population and organisation

The study included all patients newly diagnosed with HIV-1 infection as notified to the SFOPH during 01/2010-12/2012. NAT VL tests were performed at the time of HIV diagnosis in one of 11 Swiss HIV notification laboratories commissioned by the SFOPH, or at the Swiss National Center for Retroviruses (SNCR), commissioned by the SFOPH to serve as the national HIV reference laboratory. All laboratories are accredited by the governmental Swiss Accreditation Service (SAS) (http://www.seco.admin.ch/sas/index.html?lang=en).

PERT assays in patients that had exhibited ≤1’000 cp/ml of HIV-1 RNA were performed at the SNCR upon request by a patient’s treating physician. In most instances, a new blood sample was used for testing. If NAT and PERT VL were determined in two separate samples, collection dates for these samples had to be ≤100 days apart to minimize a potential decrease in VL due to initiation of ART. All repeat and alternative NAT measurements were performed on the second sample stored at the SNCR. Plasma samples were stored at −80°C and thawed once for alternative NAT and a second time for repeat NAT assessment. Measurements were performed on undiluted samples or at a maximal dilution of 1:4 in PBS.

### RNA viral load tests

Laboratories performing the initial, diagnostic NAT employed one of the following CE-marked tests: Roche COBAS AmpliPrep/COBAS TaqMan HIV-1 test v1.0 (CTM1.0), Roche COBAS AmpliPrep/COBAS TaqMan HIV-1 test v2.0 (CTM2.0), Roche COBAS Amplicor HIV-1 Monitor v1.5 (CAM1.5) or Abbott RealTime HIV-1 m2000 (ARm2000). The SNCR used CTM2.0 and ARm2000 for repeat or alternative NAT. Repeat testing with CTM1.0 and CAM1.5 was not possible, as these tests were no longer available. If possible, these samples were tested with both alternative NAT platforms (CTM2.0 and ARm2000).

### Product-enhanced-reverse-transcriptase assay

**Figure 1:**
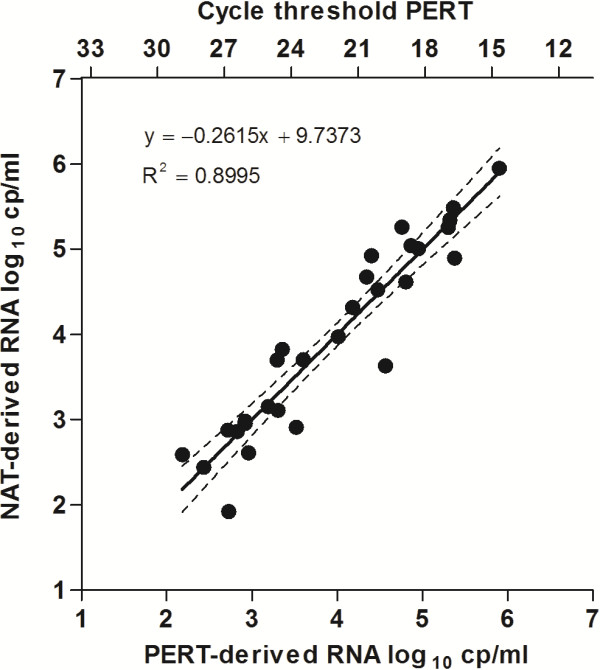
**Correlation of NAT- and PERT-derived viral loads.** NAT (CTM2.0) and PERT viral loads were determined on plasma samples of 30 untreated HIV-1 infected individuals. The top *x*-axis shows the PERT Ct-values used for the conversion to RNA log_10_ cp/ml. Dotted lines indicate the 95% confidence interval of the regression curve.

The PERT assay is an SAS-accredited method and was performed as described previously [7]. cDNA amplification and quantification was performed on the ABI7900 Sequence Detector (Perkin Elmer Biosystems, Norwalk, CT, U.S.A.). Median cycle threshold (Ct)-values were converted into RNA cp/ml based on a conversion curve generated from plasma samples of 30 untreated, HIV-1 infected patients (Figure 1). NAT VL was assessed on CTM2.0 during routine testing. The PERT assay and confirmatory NAT testing on the ARm2000 platform were performed on archived samples. The lower limit of quantification for the PERT assay was at a Ct-value of 30, which corresponds to an HIV-1 RNA concentration of 78 cp/ml.

### Duration of infection and HIV-1 subtypes

The estimated duration of HIV infection was determined by the patient’s antibody pattern in a confirmatory line immunoassay (Inno-Lia™ HIV I/II Score) [9,10]. Classification into “recent” or “older” refers to an estimated duration of infection of less or more than 120 days.

HIV-1 subtype information was obtained from the Swiss drug resistance database.

### Ethics

All data in this study were derived from diagnostic data contained in anonymised mandatory HIV notifications to the SFOPH. No informed consent was required for the anonymised notifications.

## Results

### Frequency and magnitude of low viral loads

**Figure 2:**
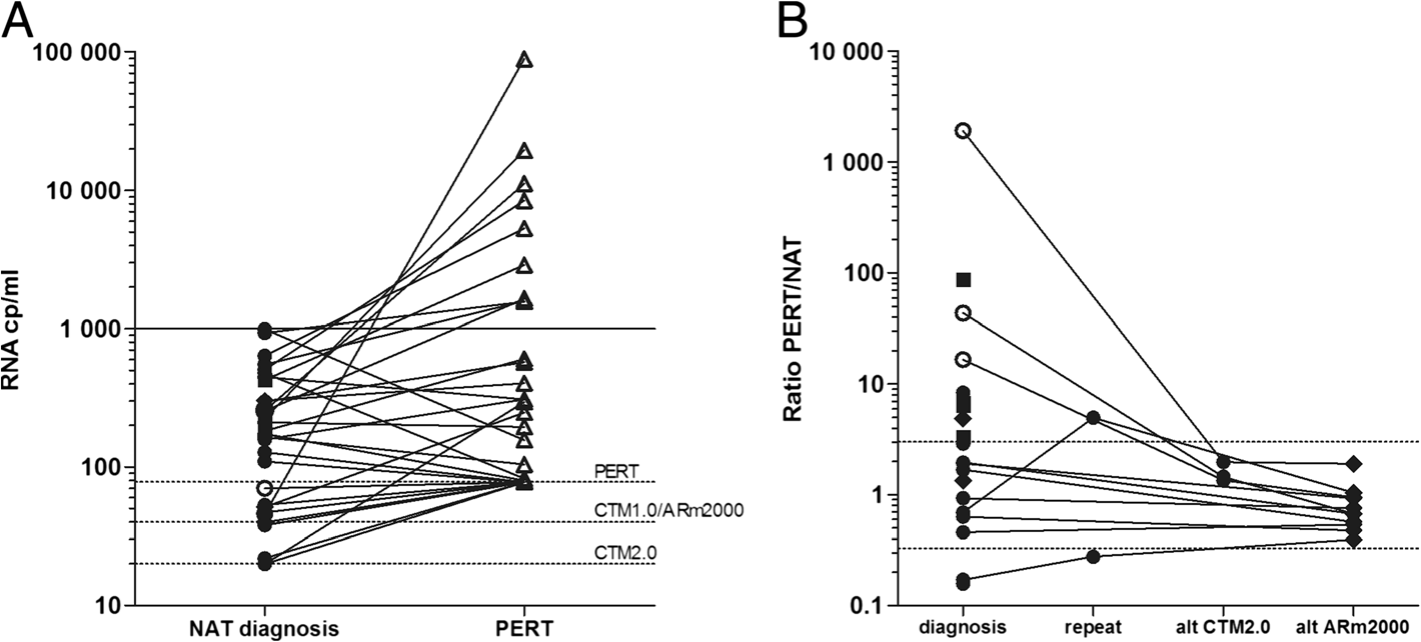
**Comparison of NAT and PERT viral loads. A**. Viral load measured by NAT and PERT at the time of diagnosis. PERT-derived viral loads were calculated based on the conversion shown in Figure 1. Samples below the limit of detection (LOD) were set to the LOD values as marked by the dotted lines. **B**. Ratio of PERT to NAT viral loads in the quantifiable range for PERT viral loads at the time of diagnosis and NAT viral loads measured at the time of diagnosis, during repeat testing and/or alternative (alt) testing with CTM2.0 or ARm2000. The area between dotted lines indicates changes in VL <3-fold. Symbols: ○ CTM1.0, ● CTM2.0, ♦ ARm2000, ■ CAM1.5, Δ PERT.

During 01/2010-12/2012, the SFOPH registered 1814 patients newly diagnosed with HIV; approximately 0.5% of these were infected by HIV-2 [11]. Seventy-one (3.9%) of the HIV-1 infected patients had an initial, diagnostic NAT VL ≤1’000 cp/ml in the absence of ART. Twelve of these (0.7% of the total) had an undetectable VL. A PERT assay was performed for 32 of the 71 patients, and the low NAT VL ≤1’000 cp/ml was confirmed in 23 patients (72% of the 32) (Table 1 and Figure 2A). The remaining 9 patients had a PERT VL >1’000 cp/ml, with seven patients (22%) exhibiting a PERT VL ≥3-fold higher than the NAT VL (patient ID 2, 4, 5, 22, 30–32 in Table 1, Figure 2B). A ≥3-fold difference in VL is considered clinically significant according to NIH guidelines [12]. In four of these patients, the VL differed by as much as 10-1’000 fold (patient ID 2, 4, 5, 30), suggesting a severe VL underestimation by the respective NAT. For all but one (patient ID 22) of the seven patients, NAT VL had been determined either with CTM1.0 (patient ID 2, 4, 5) or CAM1.5 (patient ID 30–32), i.e. with tests for which VL underestimation has been reported previously [2,13-16]. Plasma for alternative NAT assessment was available for three patients assessed using CTM1.0, and testing with the successor CTM2.0 as well as with the ARm2000 platform both confirmed a VL >1’000 cp/ml, supporting the PERT assay results (Table 1). Consequently, the ratio of PERT to NAT VL decreased to <3-fold for these patients (Figure 2B).

**Table 1:** Summary of NAT and PERT viral load results

**Patient ID**	**NAT cp/ml**				**PERT cp/ml**	**Subtype**	**Δ days samples**	**Recency**	**Year**
	**Diagnosis**	**Repeat**	**Alternative**		**Diagnosis**				
	**CTM1.0**	**CTM1.0**	**CTM2.0**	**ARm2000**					
1	TND	N/A			295	B	7	Older	2010
2	46	N/A	45‘282	46‘885	88‘733	CRF01_AE	10	Older	2010
3	70	N/A			TND		4	Older	2010
4	257	N/A	7‘728	16‘635	11‘191	B	8	Older	2010
5	508	N/A	6‘254	8‘934	8‘428	CRF02_AG	15	Older	2011
	**CTM2.0**	**CTM2.0**		**ARm2000**					
6	TND			TND	TND		4	Older	2012
7	TND				TND		14	Recent	2010
8	22				TND		19	Older	2012
9	38			TND	TND		35	Older	2012
10	47	66		54	TND		23	Older	2012
11	53				TND		9	Older	2012
12	110				TND	D	56	Older	2011
13	128			50	TND	C	24	Older	2012
14	158			459	309		22	Older	2010
15	165			220	104		10	Older	2010
16	174			148	79		20	Recent	2010
17	210			258	195	CRF02_AG	69	Older	2012
18	299			618	574	B	23	Older	2010
19	447	62		296	308		24	Older	2011
20	480	296		209	82	CRF01_AE	12	Older	2012
21	551				1‘584	CRF02_AG	20	Older	2012
22	638				5‘303	B	0	Older	2011
23	936			2‘761	1‘565		0	Older	2010
24	990				157	CRF02_AG	21	Older	2012
	**ARm2000**	**ARm2000**	**CTM2.0**						
25	TND	TND	TND		TND		77	Older	2012
26	TND		20		TND		28	Recent	2012
27	51				248		34	Recent	2010
28	302				403	B	95	Older	2010
	**CAM1.5**	**CAM1.5**							
29	182	N/A			605		3	Older	2011
30	224	N/A			19‘510	CRF02_AG	0	Older	2010
31	255	N/A			1‘642	G	21	Older	2010
32	427	N/A			2‘875	C	0	Older	2010

TND = target not detected; N/A = not applicable; Δ days = days between collection of first and second sample.

Due to the lack of sample, we could not conduct repeat or alternative NAT-measurement for the four remaining patients with ≥3-fold underestimation (patient ID 22, 30–32). However, comparison of NAT (CTM2.0 and m2000) and PERT VL in routine follow-up samples of two of these patients (patient ID 22 and 31) did not show a persistent difference in VL. Average fold difference of PERT to CTM2.0 and m2000 were 1.8 (±2.3) and 2.9 (±4.0) for patient ID 22 ( n = 7), and 5.5 and 3.0 for patient ID 31 (n = 2). This suggests technical variability or laboratory error as possible reasons for the apparent initial underestimation in these cases.

Alternative NAT measurements for all other patients (14 samples available) confirmed the low diagnostic NAT VL ≤1’000 cp/ml, indicating a genuine low VL at the time of diagnosis.

## Discussion

In this study we investigated whether commercial viral load assays in use during the past few years provided reliable concentrations of HIV-1 RNA in plasma. We focused our analysis on patients with ≤1’000 cp/ml, because the possible risks for untoward consequences of VL underestimation, namely a delay of ART or further virus transmission in a false belief of being non-infectious [17,18], are highest in this group. Confirmatory testing of the VL was possible in only 32 of the 71 patients with an initial NAT VL ≤1’000 cp/ml (45%). Possible reasons for this low rate may include the following: Usually, a new blood sample was needed for a PERT assay at the SNCR, which required the patient returning to the physician who received the information that a PERT assay was considered necessary. If the initial physician had meanwhile transferred the patient to an HIV specialist, this information may not have been forwarded to the specialist. Moreover, the decision of whether a test is performed or not ultimately remains in the physician’s responsibility; conceivable reasons for not performing a PERT assay include transient stay in the country or costs. Finally, it may not always have been possible to link a PERT result obtained for a patient sample received under full personal identity, with the coded personal information in the HIV notification.

Aside from these limitations, our analysis showed that the PERT assay confirmed the low diagnostic NAT VL in 78% of patients who had both tests. Seven patients had a clinically significant higher VL by PERT than NAT, with four patients showing a >10-fold difference. In three of these four patients we confirmed technical underestimation as the reason for the diagnostic low RNA VL, as demonstrated by significantly higher VL measurements when using alternative NAT platforms. These four most discrepant cases had all been tested with earlier NAT versions now outdated. Indeed, underestimation has been observed repeatedly for both CAM1.5 [13,15] and CTM1.0 [2,14,16] in the past. One patient showed a ≥3-fold NAT-VL underestimation on the CTM2.0 compared to PERT, which disappeared in later samples. In this case we cannot exclude some technical variance of the PERT assay.

The majority of patients were classified as having an older infection, thus minimizing concerns about large changes in VL between the first and second sample collected. Four patients classified as having a recent infection (patient ID 7, 16, 26, 27), and all had a NAT and PERT VL of ≤1’000 cp/ml. As such low VL are rarely seen in patients with recent HIV-1 infection, we cannot exclude the possibility that the diagnostic NAT VL was falsely low, but nevertheless “confirmed” by PERT because the second sample was no longer in the acute, but in the chronic phase of infection in which the VL was already at the lower set-point. We can also not exclude the possibility that the classification as recent infection was false, because the diagnostic specificity of the algorithm used in these four cases was only 93% [9]. Therefore, it is impossible to draw any safe conclusion regarding the true VL at the time of diagnosis in these four cases.

Both B and non-B subtypes were affected by NAT-based VL underestimation in our study, in agreement with others who found that underestimation was associated with a rare polymorphism in the viral genome [19]. With the introduction of CTM2.0, which uses dual target detection in gag and LTR, VL underestimation decreased dramatically [16,20,21]. The ARm2000 platform uses the IN region as target, and VL measurements performed by CTM2.0 and ARm2000 were found to correlate well [15]. Despite all technological improvement, VL underestimation may still occasionally occur even with the contemporary NAT generation [22]. This could also be the reason that one of the patients shown in Figure 1 has a PERT VL well outside the lower limit of the 95% confidence interval.

In our study population, severe NAT VL underestimation was restricted to patients for whom the initial diagnostic VL measurement was performed on NAT platforms outdated today. Although we did not find similar severe VL underestimation for any of the 23 patients assessed on either CTM2.0 or m2000, the upper limit of the 95% confidence interval of this zero frequency is at 12.2%, suggesting a certain possibility for underestimation even with contemporary viral load tests. Continuation of the rule to confirm low VL with an alternative test is thus justified from a statistical viewpoint.

## Conclusion

In light of the rapidly evolving global HIV-1 quasispecies it is virtually unavoidable that variants will arise which will be detected sub-optimally by a given sequence-based VL test. Confirmation of low VL by alternative methods like the sequence-independent PERT assay or a NAT targeting a different region of the viral genome will help to minimise such cases.

## Competing interests

The authors declare that they have no competing interests.

## Authors’ contributions

BNV analysed the data, coordinated additional sample analysis and drafted the manuscript. CS supervised routine PERT assays. JBH carried out additional sample analyses on the Abbott RealTime HIV-1 m2000 system. JB participated in the design of the study and data evaluation. JS conceived the study and participated in its design and data evaluation. All authors read and approved the final manuscript.

## Pre-publication history

The pre-publication history for this paper can be accessed here:

http://www.biomedcentral.com/1471-2334/14/84/prepub
